# Work–Family Conflict and Mental Health Among Female Employees: A Sequential Mediation Model via Negative Affect and Perceived Stress

**DOI:** 10.3389/fpsyg.2018.00544

**Published:** 2018-04-17

**Authors:** Shiyi Zhou, Shu Da, Heng Guo, Xichao Zhang

**Affiliations:** Beijing Key Laboratory of Applied Experimental Psychology, National Demonstration Center for Experimental Psychology Education (Beijing Normal University), Faculty of Psychology, Beijing Normal University, Beijing, China

**Keywords:** work–family conflict, mental health, negative affect, perceived stress, affective events theory, conservation of resources model

## Abstract

After the implementation of the universal two-child policy in 2016, more and more working women have found themselves caught in the dilemma of whether to raise a baby or be promoted, which exacerbates work–family conflicts among Chinese women. Few studies have examined the mediating effect of negative affect. The present study combined the conservation of resources model and affective events theory to examine the sequential mediating effect of negative affect and perceived stress in the relationship between work–family conflict and mental health. A valid sample of 351 full-time Chinese female employees was recruited in this study, and participants voluntarily answered online questionnaires. Pearson correlation analysis, structural equation modeling, and multiple mediation analysis were used to examine the relationships between work–family conflict, negative affect, perceived stress, and mental health in full-time female employees. We found that women’s perceptions of both work-to-family conflict and family-to-work conflict were significant negatively related to mental health. Additionally, the results showed that negative affect and perceived stress were negatively correlated with mental health. The 95% confidence intervals indicated the sequential mediating effect of negative affect and stress in the relationship between work–family conflict and mental health was significant, which supported the hypothesized sequential mediation model. The findings suggest that work–family conflicts affected the level of self-reported mental health, and this relationship functioned through the two sequential mediators of negative affect and perceived stress.

## Introduction

An old saying in China states that the husband is the breadwinner and the wife is the homemaker; this saying illustrates the clear division of gender roles in the traditional Chinese family. Based on social role expectations, women have more responsibility in taking care of the family, especially in terms of housework and children. However, in the 1950s, President Mao appealed to the masses saying that women could “hold up half of the sky,” which resulted in more and more women entering the labor force. This change led to women playing important parts in the occupational arena. The United States Bureau of Labor Statistics (2012) conducted a survey that showed the labor force participation rate of Chinese women was nearly 70%, and the labor force participation rate of Chinese women between 25 and 54 years old was up to 90%. Interestingly, both reported rates are the highest in the world. Therefore, Chinese women simultaneously play several social roles including wife, mother, daughter, daughter-in-law, as well as the role of subordinate or leader in the workplace. Since the implementation of the universal two-child policy which was published in 2016, more and more working women have found themselves caught in a dilemma of choosing between raising a family and excelling at work. Consequently, more working mothers are juggling both work and family roles. Under this circumstance, the potential influence of work–family conflict on full-time female employees has drawn increased attention from psychologists and sociologists.

Greenhaus and Beutell (1985) defined work–family conflict as a special form of inter-role conflict that arises when there are incompatible demands between work and family roles. Work–family conflict has two directions. Work-to-family conflict (WFC) occurs when experiences and commitments at work interfere with family life, and family-to-work conflict (FWC) arises when family responsibility interferes with work life. The negative consequences of work–family conflict for women and their families have been well established (Allen et al., 2000; Aryee et al., 2005; Amstad et al., 2011). Individuals with high levels of work–family conflict report more depressive symptoms (Zhang et al., 2017), more marital problems (Barling and Macewen, 1992; Higgins et al., 1992), poorer health status (Frone et al., 1997), and reduced life satisfaction, well-being, and quality of family life (Aryee et al., 1999; Stoeva et al., 2002). Therefore, a high level of work–family conflict has been associated with a variety of physical and psychological health problems. In general, these findings are indicative of a negative relationship between work–family conflict and mental health.

Mental health is a positive state or a state of well-being in which individuals can realize their abilities, cope with normal life pressures, work productively and fruitfully, and make a contribution to the community or society (World Health Organization [WHO], 2004). According to the definition of mental health, there are three core components: well-being, effective functioning in one’s personal life, and effective functioning in the community. This definition was built on two philosophical traditions: the hedonic tradition is focused on feelings of happiness, whereas the eudaimonic tradition is focused on optimal functioning in individual and social life (Waterman, 1993; Keyes, 1998). Keyes (2002) took both the hedonic and the eudaimonic approaches into account and proposed an integrated theory of positive mental health, which encompasses emotional, psychological, and social well-being. Research by Keyes et al. (2010) has demonstrated the importance of mental health. Specifically, poor mental health is closely related to future mental illness (Keyes et al., 2010), the probability of all-cause mortality (Keyes and Simoes, 2012), suicidality, academic impairment among college students (Keyes et al., 2012), and other negative outcomes. Additionally, positive mental health was related to desired workplace outcomes, such as less absenteeism (Keyes, 2007) and less unprofessional behaviors (Dyrbye et al., 2012).

Studies on mental health have also tried to identify variables that play a role in the experience of well-being. Sociodemographic variables (e.g., age, gender, and marital status), socioeconomic indicators (e.g., education and employment), and life conditions (e.g., urban/rural settings and residence) have been shown to influence well-being and mental health (Khumalo et al., 2012). Furthermore, responsibilities and expectations for family or work such as financial roles, marital roles, and parenting roles are often prescribed according to gender, which may influence the prevalence and experience of mental health (Keyes, 2002). Indeed, a significant gender difference in the relationship between work conflict and family conflict has been reported (Duxbury and Higgins, 1991). As such, conflicts between work and family life often affect mental health and these are influenced by gender.

As mentioned above, work–family conflict experienced by women working full-time has been a focus of psychological research. Many researchers have discussed the underlying factors that influence women’s work–life balance, and they have tried to find external factors to eliminate negative outcomes for women’s mental health. However, few studies have explained the mechanism of how work–family conflict impacts mental health. In the present study, we examined the role of negative affect and perceived stress in the relationship between work–family conflict and mental health.

A considerable amount of literature has examined the relationship between work–family conflict and strain over the last three decades (Bedeian et al., 1988; Allen et al., 2000; Amstad et al., 2011). The potential outcomes can be categorized into three types, which are the physiological, psychological, and behavioral reactions to environmental demands, threats, and challenges (i.e., stressors). These outcomes include responses such as health issues, well-being, and depression (Geurts et al., 2003; Greenhaus et al., 2006). Nohe et al. (2014) conducted meta-analytic path analyses on 33 longitudinal studies that had repeatedly measured WFC or FWC and strain, and results supported the common assumption that WFC/FWC predict strain.

Many researchers have considered the Conservation of Resources (COR) model as an appropriate framework for work–family studies. The COR model proposes that individuals seek to acquire and maintain resources (Hobfoll, 1989). If resources are lost or are threatened, individuals experience distress and decreased well-being. When individuals lose their identity in the process of managing both work and family roles, their resources (e.g., time, energy) gradually decline, leading to exhaustion, restlessness, and even depression. As a result, individuals experience less well-being and mental health when the demands are too high.

Extant research consistently supports that employees suffer negative consequences from work–family conflict, including decreased physical health, diminished emotional well-being and increased life distress (Frone et al., 1991; Parasuraman et al., 1996). Additionally, two meta-analyses have documented small to medium effect sizes between work–family conflict and health or well-being (Allen et al., 2000; Mesmer-Magnus and Viswesvaran, 2005). Neto et al. (2016) have used COR to analyze the development of WFC over time and have found that work–family conflict at Time One and Time Two decreased the employee psychological well-being at Time Two and Time Three, respectively. Therefore, we could infer the following:

Hypothesis 1a: WFC would be negatively related to mental health.
Hypothesis 1b: FWC would be negatively related to mental health.

In addition to the direct relationship between work–family conflict and mental health, we speculated that perceived stress and negative affect are key variables that mediate the effect of work–family conflict on mental health. Stress has become part of research literature since its introduction in 1930s. Specifically, stress was defined as a perception of acute or chronic psychological or physical pressure that causes negative changes in the individual’s body (Lyon, 2000). Cooper et al. (1987) described the conflict between work and home lives as a source of stress. According to COR model, stress is a reaction to an environment in which there is the threat or an actual loss in resources, or lack of an expected gain in resources. It follows that an individual who experience the loss of these resources, or the threat of such a loss, may therefore experience stress. The COR model explains stress outcomes for both intra- and inter-role stress, which will then lead to a negative “state of being,” including job and life dissatisfaction, depression, anxiety, and physiological tension (Grandey and Cropanzano, 1999). Therefore, it appears that work–family conflict has an indirect relationship with mental health through stress.

A number of empirical studies have reported that work–family conflict is associated with increased levels of stress (Amstad et al., 2011; Sharma et al., 2016). Additionally, some research has found that when stress was taken into consideration, work–family conflict had an indirect relationship with physical health and life distress (Frone et al., 1991; Adams et al., 1996). In particular, as a person’s resources from one role are drained so that they cannot complete another role, they may experience a negative state of being regarding both roles. Consequently, these negative states may result in their experience of a high stress level. This distress caused by stress may lead to dissatisfaction with life and to illness. Therefore, based on the literature reviewed, we could infer the following:

Hypothesis 2a: Stress would fully mediate the relationship between WFC and mental health.
Hypothesis 2b: Stress would fully mediate the relationship between FWC and mental health.

Affect refers to a mental state involving evaluative feelings (Parkinson et al., 1996). It is an umbrella term that includes a wide range of dispositions, moods, emotions, and generalized affective reactions to events, objects, and daily experiences. The construct domain of affect includes both trait-based individual differences that influence how one characteristically views and interprets the world, as well as state-based reactions that may range from somewhat transitory and specific states (e.g., moods, emotions) to more general affectively oriented evaluative judgments (e.g., job satisfaction, life satisfaction). Previous research has examined the effect of trait-based affect on work–family conflict. For instance, Stoeva et al. (2002) found that negative affect, as a theoretically and empirically independent affective trait, not only had an indirect effect on WFC through job stress and family stress, but it also moderated the effect of family stress on FWC. By contrast, the relationship between state-based affect and work–family conflict is less understood (Judge et al., 2006). Williams and Alliger (1994) found that unpleasant mood spilled over from family to work but pleasant moods had little spillover. Negative affect seems to be a critical mediator of the relationship between work–family conflict, especially for women (Rothbard, 2001; Greenhaus et al., 2006). Therefore, in the present study, we focused on the effect of state-based negative affect to investigate the subjectively experienced affective states caused by work–family conflict in order to explore the mechanism underlying the relationship between work–family conflict and outcomes.

According to Affective Events Theory (AET; Weiss and Cropanzano, 1996), specific events at work create discrete emotional reactions, which in turn lead to spontaneous work attitudes and behaviors. This theory proposes that affective experiences at work have an immediate effect such that affect influences attitudes and behaviors when an individual is in a particular affective state. This emphasis on intra-individual differences in attitudes and behavior highlights the importance of studying affective events as they unfold over time. Accordingly, we applied AET to events at home; that is, individuals’ family demands also have effects on their affective states. Based on this perspective, we assumed that if more work–family conflicts happened in women’s daily lives, they might experience more negative affect, and such negative affective experiences would have a negative influence on their work behavior and well-being. Researchers have found that state-based negative affective experiences (e.g., anxiety, tension, worry, frustration, guilt, distress, irritation) are positively related to both work–family conflict (Frone et al., 1997; Geurts et al., 2003; Matthews et al., 2006; Livingston and Judge, 2008) and greater juggling of work and family responsibilities (Williams et al., 1991; Williams and Alliger, 1994). Therefore, we could infer the following:

Hypothesis 3a: Negative affect would fully mediate the relationship between WFC and mental health.
Hypothesis 3b: Negative affect would fully mediate the relationship between FWC and mental health.

Although recent progress has been made in understanding how negative affect or perceived stress can separately influence well-being, significant gaps remain in knowledge about the relationship between negative affect and stress. Stress is concerned with unsatisfying life events that we want to change; however, affect is a basic research topic which is important for psychological and physical well -being, and social functioning (Lazarus, 2006a).

Further, affect come and go quickly with changes in circumstances (Lazarus, 2006b). Ekman and Cordaro (2011) propose open affect programs, which guide behavior automatically and involuntary. Within-subjects research has shown that negative affect is strongly correlated with perceived stress (Watson, 1988), and the stress and affect systems are tightly coupled (Montpetit et al., 2010). According to the COR model and AET, resources loss is directly related to changes in anger and depressive mood (Hobfoll, 2001), in which case employees struggling with WFC or FWC experience far more negative affect that continues to drain their physical and psychological resources. As such, perceived stress results in decreased well-being.

Physiological studies shed light on the relationship between affect and perceived stress. At a biological level, the level of cortisol is an indication of perceived stress. It has been established that hypothalamic-pituitary-adrenal (HPA) axis activity and increases in cortisol are associated with work–family imbalance (Bergman et al., 2008) and negative affect (Buchanan et al., 1999). In addition, there is increasing evidence to demonstrate that negative affect plays a mediating role in the relationship between stressful events and cortisol secretion (Van et al., 1996; Buchanan et al., 1999; Hanson et al., 2000). Based on these studies, we could infer the following:

Hypothesis 4a: Negative affect and stress would have a sequential mediating effect on the relationship between WFC and mental health.
Hypothesis 4b: Negative affect and stress would have a sequential mediating effect on the relationship between FWC and mental health.

## Materials and Methods

### Participants and Procedure

This study was approved by the institutional review board of the Beijing Normal University. Four hundred fifty-six full-time female employees in mainland China were recruited in this study through a women’s online community; however, 105 participants who worked less than 40 h per week were excluded. The average age of the participants was 30.98 (*SD* = 6.40), and 47.86% of them were mothers. They held positions across many different types of organizations (such as government departments, public institutions, state-owned enterprises, private enterprises, and foreign-funded enterprises). All participants provided informed consent online, and then they were instructed to complete a survey online, and their data was kept completely anonymous.

In the sample of 351 valid participants, 41.03% were single, 50.71% were married, and 8.26% were in other types of relationships. For length of employment, 22.79% had been working for less than 3 years, 23.08% between 3 and 5 years, 24.22% between 6 and 10 years, 24.22% between 11 and 20 years, and 5.70% more than 21 years. In terms of hours of work per week, 47.01% worked between 40 and 45 h, 25.93% between 46 and 50 h, 11.40% between 51 and 55 h, 7.41% between 56 and 60 h, and 8.26% more than 61 h. Regarding level of education, 32.19% had a degree at the junior college level or below, 53.56% had an undergraduate degree, 13.96% had a master’s degree or above, and one participant did not respond.

### Measures

#### Work–Family Conflict

Work–family conflict was measured by the Work–Family Conflict and Family–Work Conflict Scales developed by Netemeyer et al. (1996). The questionnaire consists of two subscales: WFC (Cronbach’s α = 0.89) and FWC (Cronbach’s α = 0.89). Respective example items are, “*The demands of my work interfere with my home and family life*” and “*The demands of my family or spouse/partner interfere with work-related activities*”. All 10 items are rated on a 7-point scale, ranging from 1 (*completely disagree*) to 7 (*completely agree*).

#### Negative Affect

Negative affect was measured by the Negative Affect subscale of the Positive and Negative Affect Schedule (PANAS) developed by Watson et al. (1988). The scale consists of 10 negative affect items, and respondents indicate the extent to which extent they had experienced each negative affect during the past few days on a 5-point scale ranging from 1 (*very slightly or not at all*) to 5 (*extremely*). Items such as “*Distressed*” and “*Upset.*” Cronbach’s alpha for the subscale was 0.88. The 10 items are sorted into 5 balanced parcels based on item-total correlations for inclusion as indicators of negative affect latent construct (Little et al., 2013).

#### Perceived Stress

We used the 10-item version of the Perceived Stress Scale (Cohen et al., 1983) to assess the degree to which participants experience life as stressful. Items such as “*How often have you been upset because of something that happened unexpectedly?*” and “*How often have you felt that you were unable to control the important things in your life?*” were accompanied by a 5-point scale ranging from 0 (*never*) to 4 (*very often*). Higher scores indicate greater perceived stress. Internal consistency reliability was 0.87. The 10 items are sorted into 5 balanced parcels based on item-total correlations for inclusion as indicators of the perceived stress latent construct.

#### Mental Health

Mental health was measured by the 14-item Mental Health Continuum Short Form (MHC-SF) developed by Keyes (2009), which was adapted from the 40-item Mental Health Continuum-Long Form (MHC-LF). The MHC-SF includes three components: emotional well-being (3 items), psychological well-being (6 items), and social well-being (5 items). Respective example items are “*During the past month, how often did you feel happy?*”, “*During the past month, how often did you feel that you had something important to contribute to society?*” and “*During the past month, how often did you feel that you liked most parts of your personality?*”. Respondents indicate the frequency of having experienced each feeling over the past month by rating the item from 1 (*never*) to 6 (*everyday*). Cronbach’s alpha was 0.92 for the MHC-SF full scale, and 0.89 (emotional well-being), 0.77 (social well-being), and 0.90 (psychological well-being) for the three subscales.

#### Control Variables

We controlled for participants’ age since past research has shown that negative affect decreased with age for all generations (Charles et al., 2001). Age may also have an effect on women’s perception of conflict, perceived stress, and well-being.

## Results

### Common Method Variance

Work–family conflict, negative affect, perceived stress, and mental health were measured by self-report questionnaires so there might be the problem of common method variance (CMV). We addressed this issue using Podsakoff and his colleagues’ recommendations (Podsakoff et al., 2003).

We first examined a single-factor model using Harman’s single-factor test. This test revealed a poor fit to the data in the sample (*χ^2^*/*df* = 6.106, CFI = 0.463, TLI = 0.437, RMSEA = 0.121, SRMR = 0.122). Also, we conducted an exploratory factor analysis (EFA) to check the data for work–family conflict, negative affect, perceived stress, and mental health. If only one factor emerged from the EFA or the largest one could explain most of the variance, there would be serious common method biases. The results showed that 8 factors were extracted, with the largest one accounting for 28.637% of the total variance. Second, we added an orthogonal latent common method factor to the theoretical model. The fit of this model was poor (*χ^2^*/*df* = 2.507, CFI = 0.850, TLI = 0.834, RMSEA = 0.066, SRMR = 0.186). Overall, these results suggest that common method bias was not a problem underlying the present data.

### Descriptive Statistics and Correlation Analysis

Scale means, standard deviations, and inter-correlations of the research variables are shown in **Table 1**. Hypothesis 1 predicted that WFC (1a) and FWC (1b) would be negatively related to mental health. Pearson correlation analysis was used to examine the relationships between the variables. Both women’s perceptions of WFC (*r* = -0.193, *p* < 0.01) and FWC (*r* = -0.271, *p* < 0.01) were significant negatively related to mental health. Furthermore, both WFC and FWC were significant negatively related to the three components of mental health (i.e., emotional well-being, social well-being, psychological well-being). Thus, Hypotheses 1a and 1b were supported.

**Table 1:** Descriptive statistics and correlations for the variables.

	*M*	*SD*	1	2	3	4	5	6	7
(1) WFC	3.742	1.496	–						
(2) FWC	2.411	1.317	0.430^∗∗^	–					
(3) NA	0.992	0.758	0.258^∗∗^	0.258^∗∗^	–				
(4) Stress	1.636	0.660	0.283^∗∗^	0.300^∗∗^	0.566^∗∗^	–			
(5) MH	42.920	14.347	-0.193^∗∗^	-0.271^∗∗^	-0.341^∗∗^	-0.644^∗∗^	–		
(6) EWB	8.536	3.503	-0.263^∗∗^	-0.262^∗∗^	-0.347^∗∗^	-0.561^∗∗^	0.773^∗∗^	–	
(7) SWB	15.191	5.680	-0.165^∗∗^	-0.260^∗∗^	-0.274^∗∗^	-0.511^∗∗^	0.887^∗∗^	0.567^∗∗^	–
(8) PWB	19.194	7.123	-0.129^∗^	-0.210^∗∗^	-0.298^∗∗^	-0.615^∗∗^	0.927^∗∗^	0.612^∗∗^	0.710^∗∗^

*N = 351. WFC, work-to-family conflict; FWC, family-to-work conflict; NA, negative affect; MH, mental health; EWB, emotional well-being; SWB, social well-being; PWC, psychological well-being. ^∗^*p* < 0.05, ^∗∗^*p* < 0.01 (two-tailed test).*

### Mediating Effects of Perceived Stress

We examined the fit of the models and the indirect effects by using structural equation modeling, which estimates the indirect effects of an antecedent variable (work–family conflict) on an outcome variable (mental health) via a mediator (perceived stress). In addition, we compared the model fit indices of the fully mediated model to partially mediated models. If changes in fit indices are not significant and if none of direct effect path coefficients are significant, we can accept the fully mediated model.

The fully mediated model with perceived stress as the only mediator was acceptable (*χ^2^*/*df* = 2.331, CFI = 0.916, TLI = 0.907, RMSEA = 0.062, SRMR = 0.060) and all path coefficients were statistically significant (0.227 < |*β*| < 0.760). More importantly, this fully mediated model’s fit was not significantly worse than the partially mediated model with the direct path from WFC to mental health only (Δ*χ^2^* = 0.180, Δ*df* = 1, *p* = 0.671), or the partially mediated model with the direct path from FWC to mental health only (Δ*χ^2^* = 0.449, Δ*df* = 1, *p* = 0.503). Additionally, none of the direct effect paths between work–family conflicts and mental health were significant in the partially mediated models. Considering that the fully mediated model was more parsimonious than the partially mediated models, we chose to accept the fully mediated model and reject the partially mediated models. Therefore, Hypotheses 2a and 2b were supported, and perceived stress fully mediated the relationship between work–family conflict and mental health.

### Mediating Effects of Negative Affect

We used the same data analysis procedure to examine negative affect as a mediator. The results for the fully mediated model indicated the model was acceptable (*χ^2^*/*df* = 2.272, CFI = 0.918, TLI = 0.911, RMSEA = 0.060, SRMR = 0.071). Consistent with our prediction, all path coefficients were significant (0.184 < |*β*| < 0.400) and the indirect effects of negative affect on the relationship between work–family conflict and mental health were significant. This fully mediated model’s fit was significantly worse than the partially mediated model with both direct effect paths from WFC to mental health and from FWC to mental health (Δ*χ^2^* = 11.434, Δ*df* = 2, *p* < 0.01). Based on the results of the model comparison and the significance of path coefficients, we accepted the partially mediated model with both direct effect paths (*χ^2^*/*df* = 2.254, CFI = 0.920, TLI = 0.912, RMSEA = 0.060, SRMR = 0.057), and the path coefficients between the variables were significant (except from WFC to mental health). The 95% CIs for indirect effects of negative affect in the relationship between work–family conflict and mental health were [-0.122, -0.025] and [-0.104, -0.012]. Thus, Hypotheses 3a and 3b were supported, and negative affect fully mediated the relationship between WFC and mental health, and it partially mediated the relationship between FWC and mental health.

### Sequential Mediation Analyses for Negative Affect and Perceived Stress

Taking WFC and FWC as the antecedent variables, negative affect and perceived stress as mediators, and mental health as the outcome variable, we constructed a fully mediated model, and checked the fitness of the model and significance of each path. The model fit information revealed that the fully mediated model was acceptable (*χ^2^*/*df* = 2.100, CFI = 0.918, TLI = 0.912, RMSEA = 0.056, SRMR = 0.069).

**FIGURE 1:**
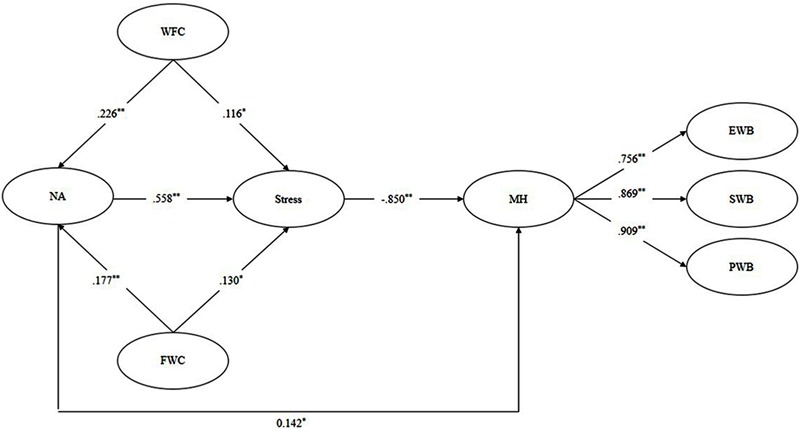
The sequential mediating effect of negative affect and perceived stress on the relationship between work–family conflict and mental health. WFC, work-to-family conflict; FWC, family-to-work conflict; NA, negative affect; MH, mental health; EWB, emotional well-being; SWB, social well-being; PWC, psychological well-being. *N* = 351. ^∗^*p* < 0.05, ^∗∗^*p* < 0.01 (two-tailed test).

Hypothesis 4a and 4b posited sequential indirect effects of negative affect and perceived stress in the relationship between work–family conflict and mental health. The path coefficients between the variables in the fully mediated model were significant (0.189 < |*β*| < 0.750). The 95% CI for sequential mediating effects of negative affect and perceived stress were [-0.172, -0.049] and [-0.150, -0.028] respectively, indicating the sequential mediating effects were significant. As present in **Table 2**, this fully mediated model fit significantly worse than partially mediated model with direct paths from work–family conflict to perceived stress and from negative affect to mental health (i.e., model 6; Δ*χ^2^* = 21.454, Δ*df* = 3, *p* < 0.01). Considering the results of the model comparisons and the significance of path coefficients, we accepted the model 6 (*χ^2^*/*df* = 2.073, CFI = 0.921, TLI = 0.914, RMSEA = 0.055, SRMR = 0.057), and all path coefficients were significant (0.116 < |*β*| < 0.850, see **Figure 1**). The 95% CIs for sequential mediating effects of negative affect and perceived stress were [-0.172, -0.043] and [-0.146, -0.021] respectively, indicating that the sequential mediating effects were significant (see **Table 3**). Therefore, Hypothesis 4a and 4b were supported, and negative affect and perceived stress appear to have sequential, fully mediating effects, in the relationship between work–family conflict and mental health.

**Table 2:** Model comparisons about the fully mediated model and partially mediated models for the sequential mediating effects.

Model	*χ^2^*	*df*	Δ*χ^2^*	CFI	TLI	RMSEA	SRMR
*Model 0:* sequential fully mediated model	1155.177	550		0.918	0.912	0.056	0.069
*Model 1:* partially mediated model with path WFC→MH	1155.169	549	0.008	0.918	0.911	0.056	0.068
*Model 2:* partially mediated model with path FWC→MH	1153.775	549	1.402	0.918	0.911	0.056	0.065
*Model 3:* partially mediated model with path WFC→stress	1143.565	549	11.612^∗∗^	0.920	0.913	0.056	0.061
*Model 4:* partially mediated model with path FWC→stress	1142.667	549	12.510^∗∗^	0.920	0.913	0.056	0.058
*Model 5:* partially mediated model with path NA→MH	1150.629	549	4.548^∗^	0.919	0.912	0.056	0.070
*Model 6:* partially mediated model with paths WFC→stress, FWC→stress, and NA→MH	1133.723	547	21.454^∗∗^	0.921	0.914	0.055	0.057

*WFC, work-to-family conflict; FWC, family-to-work conflict; NA, negative affect; MH, mental health. ^∗^*p* < 0.05, ^∗∗^*p* < 0.01 (two-tailed test).*

**Table 3:** Indirect effects for the sequential mediating effects.

Path	Standardized coefficient	95% CI Lower	95% CI Upper
WFC→NA→stress	0.126	0.054	0.199
WFC→NA→MH	0.032	-0.002	0.067
WFC→stress→MH	-0.099	-0.193	-0.005
WFC→NA→stress→MH	-0.107	-0.172	-0.043
FWC→NA→stress	0.099	0.027	0.170
FWC→NA→MH	0.025	-0.004	0.054
FWC→stress→MH	-0.110	-0.204	-0.017
FWC→NA→stress→MH	-0.084	-0.146	-0.021
NA→stress→MH	-0.474	-0.582	-0.366

*WFC, work-to-family conflict; FWC, family-to-work conflict; NA, negative affect; MH, mental health.*

As shown in **Table 3**, the results of separated mediation effects of negative affect and perceived stress were not consistent with the previous analyses. The indirect effects of negative affect in the relationship between work–family conflict and mental health were not significant, but the indirect effects of perceived stress were significant (*p* < 0.05). The 95% CIs for the indirect effects of negative affect were [-0.002, 0.067] and [-0.004, 0.054] respectively, and the 95% CIs for the indirect effects of perceived stress were [-0.193, -0.005] and [-0.204, -0.017] respectively.

## Discussion

Although the effects of women’s work–family conflict on well-being have been well-documented, the role of negative affect and perceived stress in this relationship has been somewhat inconclusive. In the present study, we used a sample of full-time female employees and attempted to uncover how both negative affect and perceived stress are related to self-reported mental health. The results support the important roles that negative affect and perceived stress play as mediators in the relationship between work–family conflict and mental health. Work–family conflict was a predictor of mental health not only through negative affect and perceived stress separately, but also through a sequential mediating effect of negative affect and perceived stress. The results support the integrated model of work–family conflict, and further indicate the directions for improving women’s well-being.

These results are consistent with AET theory and the COR model, which posit that employees’ behaviors or perceptions are influenced by events, objects, or daily experiences. On one hand, affective experiences have an immediate effect on attitudes or behaviors. As such, if a female employee experiences WFC or FWC, she will be more likely to feel unhappy, which influences her perception of well-being. These results are consistent with Eby et al. (2010) who proposed that affective experiences appear to be an important explanatory mechanism in the work and family interaction. On the other hand, if female employees perceive stress due to devoting their energy or time to WFC or FWC, women may come to believe that they cannot be happy or enjoy their life. As such, if female employees juggle work and family roles, they will feel the loss of resources and they may also feel stressed, and this combination leads to worse mental health. Sharma et al. (2016) demonstrated that work–family conflicts led to stress among nursing staff, which subsequently had an impact on their psychological health. The present study obtained results that are consistent with this past research, though the present study examined these findings in a different population.

We also found support for the sequential mediating effects of negative affect and perceived stress. Female employees’ work–family conflict was related to their self-rated mental health, and this relationship was mediated by their negative affect and perceived stress, which is consistent with what has been conjectured in prior research on work stress (Hanson et al., 2000). It is understandable that WFC and FWC could lower mental health through negative affect or perceived stress separately. Similarly, these relationships may function through both negative affect and stress. Furthermore, the direct effects were not significant, indicating that negative affect and perceived stress explained the relationships.

Sequential mediation analyses were conducted to examine the roles of negative affect and perceived stress in the relationships between work–family conflict and mental health. The results revealed that negative affect mediated the relationship between WFC/FWC and perceived stress but not the relationship between WFC/FWC and mental health; this finding was inconsistent with the results in the analyses for separate mediation effects of negative affect. It is possible that the reason for insignificant indirect effects of negative affect was the relationship between negative affect and perceived stress. Prior studies indicate that negative affect strongly correlates with perceived stress, and these systems tightly coupled (Watson, 1988; Montpetit et al., 2010). Considering that the relationship between positive and negative affect appears to be independent (Diener and Emmons, 1984), another possible reason was that emotional well-being subscale was measured by three positive affect items. This measurement may have influenced the estimation of path coefficient from negative affect to mental health. Therefore, it will not impair women’s mental health until they suffer from stressful experience caused by work–family conflict and negative affect.

### Theoretical Implications

There are at least four reasons why the results of the present study are considered theoretically important. First, the present study highlights the effect of affect in the relationship between work–family conflict, stress, and mental health. Previous studies have examined the relationship between work–family conflict, stress, and some negative outcomes through the model of stressor-stress-strain. However, the effect of affect in this interaction has not been fully understood. Some studies have treated affect as the outcome of stress, which overlooks the possibility that affects may play a role in the functional mechanism. Therefore, our study complements the stressor-stress-strain model by explaining the mediating effect of negative affect.

Second, the present study examined multiple mediation models to understand the relationship between work–family conflict and mental health. If future research replicates these results, it suggests that work–family conflict leads women to experience negative affect, and the accumulation of negative affective experiences leads to stress, which impairs well-being. This argument suggests that future researchers should conduct experiments to explore the sequence of negative affect and perceived stress.

Third, there has been considerable debate as to whether negative affect or perceived stress explains more of the variance in this relationship between work–family conflict and mental health. From the results of our sequential mediation model, we propose that the relationship between conflict and well-being may be more complex than previously thought. Specifically, we found that both negative affect and perceived stress can function as mediators in this relationship and perceived stress may play a larger role. These results suggest that both directions of conflict (WFC and FWC) are related to mental health, and the researcher should not conflate stressors and perceived stress.

Finally, the most important contribution made by the present study is the examination of the sequential mediating effects of negative affect and perceived stress. Although past research has claimed that negative affect and perceived stress play critical roles in determining employees’ well-being, empirical investigations of these important variables have been rather rare and incomplete. We found that the relationship between work–family conflict and mental health was mediated by negative affect and stress. Specifically, this kind of relationship could be mediated respectively or successively.

### Practical Implications

From a practical perspective, our study may provide some direction for organizational and individual interventions focused on the harmful effects of work–family conflict on mental health. Coping is an integral part of the process of emotional arousal and stress. The three concepts, (i.e., stress, affect, and coping), work together and form a conceptual unit, with emotion being the superordinate concept (Lazarus, 2006b). Therefore, this study indicates that in order to decrease the influence of work–family conflict on mental health, women should focus on negative affect regulation, and stress management. When work–family conflicts happened to full-time female employees, they should first realize what consequence it has caused to their affect, especially negative affect, and find ways to cope. In other words, working women are encouraged to evaluate their stress levels and reduce their stress through useful methods. For instance, mindfulness based stress reduction (MBSR), acceptance and commitment therapy (ACT) and rational-emotive therapy (RET).

For organizations, leaders and Human Resources workers can provide training in emotion regulation to their employees to help them manage work–family conflicts and stress better, which may improve their level of happiness. For individuals, our study suggests that it is important to be aware of one’s emotions, especially negative affect, and to learn skills to reduce or alleviate negative affect and manage stress.

### Limitations and Future Research

The present study is the first study that has examined a sequential mediation model of the association between work–family conflict and self-reported mental health. However, there are several limitations that should be noted.

First, in order to better understand the relationship between work–family conflict and affect, our study focused on negative affect as a mediator, as previous studies have argued that affect has a two-dimensional structure consisting of positive and negative affect (Feldman Barrett and Russell, 1998; Stanley and Burrows, 2001). Positive affect refers to the experience of pleasant psychological states such as cheerfulness, alertness, and confidence. Negative affect refers to the experience of unpleasant psychological states such as anger, sadness, and fear (Gray and Watson, 2001). Findings from the work–family conflict perspective are associated with multiple demands, which are detrimental to individuals and invoke negative emotional responses and strain. On the contrary, work–family enrichment perspective suggests that there are benefits of role involvement which lead to gratification and positive affect (Rothbard, 2001). Future research may need to focus on the relationship between work–family enrichment and positive affect, and then explore the potential role of positive affect as a mediator.

Second, this study focused on the negative outcomes of work–family conflict and its process. An extant meta-analysis suggests that there are reciprocal effects of WFC/FWC and strain (Nohe et al., 2014). As such, research could extend this model by taking reciprocal effects into account, and provide empirical evidence about how WFC and FWC are related to strain.

Third, our study used a cross-sectional research design, which cannot provide adequate support for causal relationships between variables. Compared to longitudinal design, cross-sectional designs may bias the estimation of mediation parameters even when mediation is complete (Maxwell and Cole, 2007). Although research has shown a strong relationship between negative affect and perceived stress (Paukert et al., 2006; O’Hara et al., 2014; Krieger et al., 2015), the results of present study support that negative affect is the antecedent variable of perceived stress. It is unclear whether negative affect causes perceived stress or perceived stress causes negative affect. Future research may consider using a longitudinal design or daily diary methods to test the causal relationship between negative affect and perceived stress.

Forth, our study relied only on self-report measures. Multiple methods of assessment should be considered in future studies. For example, given that the level of cortisol is a signal of perceived stress, future research could include physiological measurements.

## Conclusion

The present study suggests that there is a significant sequential mediating effect of negative affect and perceived stress in the relationship between work–family conflict and mental health among Chinese female employees who work full-time. Therefore, to improve Chinese female employees’ mental health as a consequence of work–family conflict, it would be important to increase emotion regulation and stress management skills.

## Author Contributions

SZ designed the study and collected the data. SZ and SD performed the data analysis and wrote the manuscript, so worked as co-first authors. XZ was the supervisor of the research team, and he repeatedly revised the manuscript with HG. All authors contributed to this work and approved the final version of the manuscript to be published.

## Conflict of Interest Statement

The authors declare that the research was conducted in the absence of any commercial or financial relationships that could be construed as a potential conflict of interest.
